# Association Between Maternal Prepregnancy Body Mass Index and Anthropometric Parameters, Blood Pressure, and Retinal Microvasculature in Children Age 4 to 6 Years

**DOI:** 10.1001/jamanetworkopen.2020.4662

**Published:** 2020-05-12

**Authors:** Bianca Cox, Leen J. Luyten, Yinthe Dockx, Eline Provost, Narjes Madhloum, Patrick De Boever, Kristof Y. Neven, Franco Sassi, Hanne Sleurs, Karen Vrijens, Paolo Vineis, Michelle Plusquin, Tim S. Nawrot

**Affiliations:** 1 Center for Environmental Sciences, Hasselt University, Hasselt, Belgium; 2 Unité de Recherche en Biologie Cellulaire, Namur Research Institute for Life Sciences, Namur University, Namur, Belgium; 3 Health Unit, Flemish Institute for Technological Research, Mol, Belgium; 4 Imperial College Business School, Centre for Health Economics and Policy Innovation, London, England; 5 School of Public Health, Imperial College, London, England; 6 Environment & Health Unit, Department of Public Health, Leuven University, Leuven, Belgium

## Abstract

**Importance:**

Maternal prepregnancy body mass index (BMI; calculated as weight in kilograms divided by height in meters squared) has previously been associated with offspring cardiometabolic risk factors, such as fat mass, glucose and insulin levels, and blood pressure, but these associations appear to be largely mediated by offspring BMI. To our knowledge, no studies have assessed alterations in the retinal microvasculature in association with maternal prepregnancy BMI.

**Objective:**

To investigate the association between maternal prepregnancy BMI and anthropometric parameters, blood pressure, and retinal vessel parameters in children age 4 to 6 years.

**Design, Setting, and Participants:**

Participants included mother-child pairs of the population-based Environmental Influence on Early Aging (ENVIRONAGE) birth cohort study (Flanders, Belgium) who were recruited at birth from February 2010 to June 2014 and followed-up at age 4 to 6 years between October 2014 and July 2018. Data were analyzed from February 2019 to April 2019.

**Exposures:**

Maternal prepregnancy BMI based on height and weight measurements at the first antenatal visit (weeks 7-9 of gestation).

**Main Outcomes and Measures:**

Children’s anthropometric, blood pressure, and retinal microcirculation measurements at age 4 to 6 years. Retinal vessel diameters and the tortuosity index, a measure for the curvature of the retinal vasculature, were obtained by fundus image analysis.

**Results:**

This study included 240 mothers and children with a mean (SD) age of 29. 9 (4.2) years and 54.8 (4.7) months, respectively. Of these, 114 children (47.5%) were boys. Maternal prepregnancy BMI was positively associated with the child’s birth weight, BMI, waist circumference, blood pressure, and retinal vessel tortuosity. A 1-point increase in maternal prepregnancy BMI was associated with a 0.26–mm Hg (95% CI, 0.08-0.44) higher mean arterial pressure for their children, with similar estimates for systolic and diastolic blood pressure. Independent from the association with blood pressure, a 1-point increase in maternal prepregnancy BMI was associated with a 0.40 (95% CI, 0.01-0.80) higher retinal tortuosity index (× 10^3^). The hypothesis that these associations reflect direct intrauterine mechanisms is supported by the following observations: associations were independent of the current child’s BMI and the estimates for paternal BMI at the follow-up visit did not reach significance.

**Conclusions and Relevance:**

Considering that blood pressure tracks from childhood into adulthood and microvascular changes may be early markers of cardiometabolic disease development, our results suggest that maternal prepregnancy BMI is an important modifiable risk factor for later-life cardiovascular health of the offspring.

## Key Points

**Question:**

Is maternal prepregnancy body mass index (BMI) associated with anthropometric, blood pressure, and retinal vessel parameters in children age 4 to 6 years?

**Findings:**

In this birth cohort study of 240 mother-child pairs, mothers with a higher prepregnancy BMI had children with a significantly higher blood pressure and retinal vessel tortuosity at age 4 to 6 years. These associations were independent of the child’s BMI.

**Meaning:**

Interventions aiming at a healthy BMI from preconception onwards might improve the cardiovascular health of the next generation.

## Teaser

This cohort study examines the the association between maternal prepregnancy body mass index and anthropometric parameters, blood pressure, and retinal vessel parameters in Flemish children age 4 to 6 years.

## Introduction

The increasing prevalence of obesity is a global public health problem. Estimates suggest that more than 21% of women will have obesity by 2025,^1^ which is concerning considering that obesity during pregnancy is not only associated with adverse maternal and fetal pregnancy outcomes^2^ but also with potential long-term health effects in the context of the developmental origins of health and disease theory^3^ and molecular longevity of the next generation.^4^ Maternal prepregnancy obesity is associated with adverse childhood cardiometabolic, respiratory, and cognitive-related health outcomes^5^ as well as with increased cardiometabolic morbidity and mortality in their adult offspring.^6,7^ Although the mechanisms underlying these associations remain unclear, direct intrauterine effects, as well as shared genetic, environmental, or lifestyle factors, may be involved.^5^

Identifying blood pressure determinants in children is highly relevant given that blood pressure tracks from childhood into adulthood^8^ and elevated blood pressure in adult life is a leading risk factor for cardiovascular disease (CVD).^9,10^ A recent review concluded that the evidence for an association between maternal prepregnancy body mass index (BMI; calculated as weight in kilograms divided by height in meters squared) and offspring’s blood pressure is limited and that this association is mainly mediated by offspring’s anthropometry. The authors also concluded that good-quality studies on the association between maternal prepregnancy BMI and offspring’s mean arterial pressure are lacking.^11^

Although the microcirculation makes up most of the circulatory system, it is often ignored in studies assessing cardiometabolic phenotypes. Retinal vascular imaging is a noninvasive tool to study the human microcirculation in vivo. Changes in retinal vessel diameter have been proposed as early markers of cardiometabolic disease development as they have been associated with health outcomes, such as obesity,^12^ diabetes,^13^ hypertension,^14^ coronary heart disease,^15,16^ and stroke.^17,18^ Increased retinal vessel tortuosity has been associated with disease conditions, such as diabetic retinopathy^19^ and ischemic stroke,^20^ in adult populations. To our knowledge, no studies have examined the association between maternal prepregnancy BMI and the retinal microvasculature of the offspring.

In this study, we investigated the association between maternal prepregnancy BMI and offspring cardiometabolic outcomes (birth weight, BMI, waist circumference, blood pressure, and retinal microcirculation parameters) at age 4 to 6 years and assessed how the associations with blood pressure and retinal parameters were explained by child’s anthropometry. We also explored whether the observed associations reflect a shared familial lifestyle instead of intrauterine mechanisms by comparing estimates for maternal prepregnancy BMI with estimates for maternal and paternal BMI at the time of offspring cardiometabolic measurements.

## Methods

### Study Population

Within the framework of the Belgian birth cohort Environmental Influence on Early Aging (ENVIRONAGE) study, mother-newborn pairs were recruited on arrival for delivery at the East-Limburg Hospital in Genk, Belgium.^21^ Recruitment was conducted according to the Helsinki Declaration^22^ and procedures were approved by the ethical committees of Hasselt University and the East-Limburg Hospital. Mothers with a singleton pregnancy who were able to complete a questionnaire in Dutch were eligible for participation. At age 4 to 6 years, mother-child pairs were invited for a follow-up examination, which was conducted in a well-equipped, child-friendly research room located at Hasselt University– Diepenbeek. Before the clinical examinations began, mothers renewed the written informed consent they provided at recruitment and children gave their assent for the measurements. It was indicated to mothers and children that measurements were not obligatory and could be stopped at any moment. Recruitment started in February 2010 and follow-up visits in October 2014. The participation rate of the follow-up phase was 56.6%. At the end of July 2018, follow-up data were available for 319 children who were born during weekends before July 2014. Children with missing data on retinal microcirculation (62 [19.4%]), blood pressure (13 [4.1%]), or maternal BMI measured at the follow-up visit (3 [0.9%]) were excluded from the analysis. One child (0.3%) with an outlying BMI value (9.9) at follow-up was also excluded, resulting in a final sample size of 240.

Information on newborns’ sex and birth weight, gestational age, maternal age, maternal prepregnancy weight, height, and weight before delivery was retrieved from the medical hospital records. Gestational age was estimated based on the mother’s last menstrual period in combination with ultrasonography data. Maternal height and weight were measured at the first antenatal visit (weeks 7-9 of gestation) wearing no shoes and light clothes. Prepregnancy BMI was categorized into 4 groups: underweight (less than 18.5), normal weight (18.5–24.9), overweight (25.0–29.9), and obese (30.0 or more). Maternal pregnancy weight gain was calculated from the prepregnancy weight and the weight measured on admission to the delivery ward.

Detailed information about sociodemographic and lifestyle factors, such as parity, the newborn’s race/ethnicity, maternal education, and maternal smoking during pregnancy was obtained from questionnaires completed after delivery. Parity was categorized into 1, 2, and 3 or more children. Classification of race/ethnicity was based on the native country of the neonates’ grandparents as either European (at least 2 grandparents were European) or non-European (at least 3 grandparents were of non-European origin). The categories for maternal educational level were no diploma (low education), high school diploma (medium education), and college or university degree (high education). Maternal smoking status was defined as never smoked, stopped smoking before pregnancy, and smoked during pregnancy. Data on paternal height and weight were obtained from a questionnaire administered at the follow-up visit.

### Clinical Measurements at the Follow-up Visit

Anthropometric, blood pressure, and retinal microcirculation measurements were performed by a trained examiner in a quiet environment. The weight of mothers and children was measured to the nearest 0.1 kg with a digital scale and the height with a fixed stadiometer with an accuracy of 0.5 cm. The waist circumference (WC) of the child was determined at the level of the umbilicus to the nearest 0.1 cm.

Children’s blood pressure was measured using the automated Omron 750IT (HEM-759-E) oscillometric device previously validated in children.^23^ According to a standard protocol,^24^ blood pressure measurements were taken on the right arm while children were seated with the upper arm at heart level. To ensure an accurate measurement, appropriate cuffs for children in sizes small (15-22 cm) or extra small (9-14 cm) were used depending on the child’s arm circumference. Five consecutive measurements were taken with 1-minute intervals. Systolic blood pressure (SBP) and diastolic blood pressure (DBP) were calculated by averaging the last 3 measurements. Mean arterial pressure was defined as (2DBP + SBP)/3.

Images of the fundus of the left and right eye of the child were taken with a Canon CR-2 plus 45° 6.3 megapixels digital nonmydriatic retinal camera (Hospithera) and were analyzed with the semiautomated retinal vessel analysis software (MONA-REVA, version 2.1.1; VITO Health). The software analyses the central retinal arteriolar equivalent (CRAE) and the central retinal venular equivalent (CRVE), according to the revised Parr-Hubbard formula.^25^ These are the composite average retinal arteriolar and venular diameters based on the 6 widest arterioles and venules, respectively, that were identified within an area equal to 0.5 to 1 disc diameter from the optic disc (OD) margin. The arteriovenous ratio (AVR) is the ratio between CRAE and CRVE. The tortuosity index (TI), calculated as an average tortuosity of arterioles and venules, is a measure for the curvature of the retinal vasculature. The TI is computed as the average tortuosity of the branch segments in which the tortuosity of a branch segment is the ratio of the line traced on each tree along the vessel axis between 0.5 and 2 times the OD diameter and the line connecting the end points.^26^ Segmentations are cropped centered on the OD, whereby the inner and outer radii were taken at 1.5 and 5.0 times the radius of the OD. The CRAE and CRVE measurements were validated in a group of 61 healthy individuals between age 22 to 56 years by photographing the fundus of the right eye twice within a period of 5 minutes on 4 consecutive days at approximately the same time.^27^ When validating the tortuosity values on the publicly available RET-TORT data set, a Spearman rank correlation coefficient of 0.88 was obtained (unpublished data). Average CRAE, CRVE, and TI values of both eyes were used in further analyses if both pictures were available. For 44 children (18.3%) included in the analysis, values from a single eye were used.

### Statistical Analysis

Data were analyzed from February 2019 to April 2019 using R (version 3.5.2; R Development Core Team). Associations between maternal prepregnancy BMI and offspring cardiometabolic outcomes (birth weight, BMI, WC, SBP, DBP, mean arterial pressure [MAP], CRAE, CRVE, AVR, and TI) were investigated with linear regression models. Base models were adjusted for sex, gestational age, parity, newborn race/ethnicity, maternal age, maternal education, maternal smoking, gestational weight gain, date of follow-up visit (linear term), season of follow-up, and age of the child at follow-up (in months). Base models for retinal microcirculation measurements (CRAE, CRVE, AVR, and TI) were additionally adjusted for MAP, and the model for CRAE was additionally adjusted for CRVE and vice versa. To assess whether these associations were driven by child’s anthropometry, the next set of models was additionally adjusted for child’s birth weight and BMI. Because some evidence for nonlinearity was observed in the associations of child’s birth weight and BMI with child’s blood pressure and retinal parameters, these variables were modeled with a natural cubic spline with 3 df.

We also investigated associations of maternal and paternal BMI at the follow-up visit with offspring cardiometabolic endpoints (in separate models). Stronger associations for maternal prepregnancy BMI than for maternal or paternal BMI at follow-up support the existence of an intrauterine mechanism (rather than confounding by shared familial exposures). For the comparison of estimates for maternal prepregnancy BMI and paternal BMI at follow-up, models for prepregnancy BMI were rerun on the sample with available data for paternal BMI (214 [89.2%]).

In a sensitivity analysis, potential nonlinearity in the associations between maternal prepregnancy BMI and offspring cardiometabolic outcomes was investigated by modeling maternal BMI using a natural cubic spline with 3 *df*. Statistical significance was set at *P* < .05 and all tests were 2-tailed. This study was performed according to the Strengthening the Reporting of Observational Studies in Epidemiology (STROBE) reporting guidelines for observational studies.^28^

## Results

General characteristics of our study population (240 participants) are provided in Table 1. Twelve of the 240 children (5.0%) were born preterm (gestational age younger than 37 weeks). At the first antenatal visit (prepregnancy BMI), 8 mothers (3.3%) had underweight, 51 (21.3%) had overweight, and 28 (11.7%) had obesity. At the follow-up visit, six mothers (2.5%) had underweight, 59 (24.6%) had overweight, and 18 (7.5%) had obesity. Of the 214 fathers with available BMI data, 2 (0.9%) had underweight, 91 (42.%) had overweight, and 27 (12.6%) had obesity. Characteristics such as maternal age, parity, newborn’s sex, gestational age, and birth weight of the ENVIRONAGE sample were similar to those of all deliveries in Flanders, Belgium^29^ (eTable 1 in the Supplement) except for the higher proportion of highly educated mothers (66.3% in the study sample vs 46.1% in Flanders) and newborns of European descent (93.8% vs 87.7%).

**Table 1.** Characteristics of the Study Population (N = 240)

Characteristic	Participants, No. (%)
**Birth**	
Boys	114 (47.5)
Gestational age, mean (SD), wk	39.2 (1.4)
Birth weight, mean (SD), g	3445 (431)
Parity	
1	122 (50.8)
2	91 (37.9)
≥3	27 (11.3)
European race/ethnicity^a^	225 (93.8)
Maternal age, mean (SD), y	29.9 (4.2)
Maternal educational level^b^	
Low	17 (7.1)
Medium	64 (26.7)
High	159 (66.3)
Maternal smoking status	
Never smoked	161 (67.1)
Stopped before pregnancy	46 (19.2)
Smoked during pregnancy	33 (13.8)
Maternal prepregnancy BMI, mean (SD)	24.3 (4.6)
Gestational weight gain, mean (SD), kg	13.7 (5.0)
**Follow-up visit**	
Season	
Winter	71 (29.6)
Spring	79 (32.9)
Summer	46 (19.2)
Autumn	44 (18.3)
BMI, mean (SD)	
Maternal	24.2 (4.0)
Paternal^c^	25.8 (3.4)
Child, mean (SD)	
Age, mo	54.8 (4.7)
BMI	16.1 (1.4)
Waist circumference, cm^d^	53.6 (4.0)
SBP, mm Hg	97.6 (8.0)
DBP, mm Hg	54.0 (6.8)
MAP, mm Hg	68.5 (6.0)
CRAE, μm	180.9 (14.3)
CRVE, μm	250.8 (19.4)
AVR, %	72.3 (5.0)
TI × 10^3^	889 (13)

Abbreviations: AVR, arteriole-to-venule ratio; BMI, body mass index (calculated as weight in kilograms divided by height in meters squared); CRAE, central retinal arteriolar equivalent; CRVE, central retinal venular equivalent; DBP, diastolic blood pressure; MAP, mean arterial pressure; SBP, systolic blood pressure; TI, tortuosity index.

^a^ Newborns were classified as European when at least 2 grandparents were European and non-European when at least 3 grandparents were of non-European origin.

^b^ Maternal educational level was coded low when mothers did not obtain any diploma, medium when they obtained a high school diploma, and high when they obtained a college or university degree.

^c^ N = 214.

^d^ N = 237.

The motivation to correct for a child’s birth weight and BMI by using nonlinear functions is presented in eFigures 1 (blood pressure parameters) and 2 (retinal microcirculation parameters) in the Supplement. We did not find evidence for associations between children’s birth weight and cardiovascular measurements except for the U-shaped exposure-response curve for CRVE and the nonlinear curve for AVR, indicating a lower AVR for high birth weights. Nonlinear curves were also observed for DBP and CRVE in association with children’s BMI. Diastolic blood pressure was higher for children with a low BMI, whereas CRVE was higher for children with a high BMI.

Maternal prepregnancy BMI was positively associated with children’s anthropometric outcomes (birth weight, BMI, and WC), children’s blood pressure (SBP, DBP, MAP), and retinal TI, but no significant associations were observed for CRAE, CRVE, and AVR (Table 2). Results were similar after adjustment for children’s birth weight and BMI (Table 2). A 1-point increase in higher maternal prepregnancy BMI was associated with a 0.27–mm Hg (95% CI, 0.03-0.51) higher SBP, a 0.26–mm Hg (95% CI, 0.06-0.45) higher DBP, a 0.26–mm Hg (95% CI, 0.08-0.44) higher MAP, and a 0.40 (95% CI, 0.01-0.80) higher TI × 10^3^ in their offspring.

**Table 2.** Associations Between Maternal Prepregnancy BMI and Child Cardiometabolic End Points^a^

Characteristic	Base model		Fully adjusted model	
	Estimate (95% CI)	*P *value	Estimate (95% CI)	*P *value
Birth weight, g	11.5 (0.7 to 22.2)	.04	NA	NA
BMI	0.08 (0.04 to 0.12)	<.001	0.08 (0.04 to 0.12)	<.001
WC, cm^b^	0.16 (0.05 to 0.27)	.01	0.14 (0.03 to 0.25)	.01
SBP, mm Hg	0.35 (0.12 to 0.57)	.003	0.27 (0.03 to 0.51)	.03
DBP, mm Hg	0.18 (−0.01 to 0.36)	.07	0.26 (0.06 to 0.45)	.01
MAP, mm Hg	0.23 (0.07 to 0.40)	.01	0.26 (0.08 to 0.44)	.004
CRAE, μm	−0.19 (−0.54 to 0.15)	.27	−0.18 (−0.55 to 0.18)	.33
CRVE, μm	0.29 (−0.18 to 0.75)	.23	0.31 (−0.17 to 0.79)	.21
AVR, %	−0 10 (−0.25 to 0.05)	.19	−0.10 (−0.26 to 0.06)	.22
TI x 10^3^	0.32 (−0.05 to 0.69)	.09	0.40 (0.01 to 0.80)	.045

Abbreviations: AVR, arteriole-to-venule ratio; BMI, body mass index (calculated as weight in kilograms divided by height in meters squared); CRAE, central retinal arteriolar equivalent; CRVE, central retinal venular equivalent; DBP, diastolic blood pressure; MAP, mean arterial pressure; NA, not applicable; SBP, systolic blood pressure; TI, tortuosity index; WC, waist circumference.

^a^ Estimates represent the difference (with 95% CIs) in child cardiometabolic outcomes associated with an increase of 1 in maternal prepregnancy BMI and were adjusted for sex, gestational age, parity, newborn race/ethnicity, maternal age, maternal education, maternal smoking, gestational weight gain, date and season of follow-up visit, and children’s age. Estimates for retinal microcirculation parameters were additionally adjusted for MAP and estimates for CRAE were additionally adjusted for CRVE and vice versa. Estimates from fully adjusted models were additionally adjusted for children’s birth weight (except for the estimate for birth weight) and BMI (except for the estimates for BMI and WC). Children’s birth weight and BMI were modeled using a natural cubic spline with 3 df.

^b^ N = 237 (n = 240 for other models).

For child blood pressure parameters, associations with maternal BMI measured at the follow-up visit were similar to the associations observed for prepregnancy BMI, with slightly higher estimates for the latter (Figure). For children’s TI, the estimate for maternal BMI at follow-up was not significant and considerably lower than the estimate for prepregnancy BMI. However, for children’s other retinal parameters, associations with maternal BMI at follow-up appeared to be stronger than associations with prepregnancy BMI, with significant estimates for CRVE and AVR. The estimated change in child CRVE and AVR for a 1-point increase in higher maternal BMI at follow-up was 0.63 μm (95% CI, 0.11-1.14 μm) and −0.18% (95% CI, −0.35 to −0.01%), respectively. Associations with paternal BMI were mostly weaker than associations with maternal prepregnancy BMI and only the estimate for child’s BMI was significant (Figure).

**Figure.**
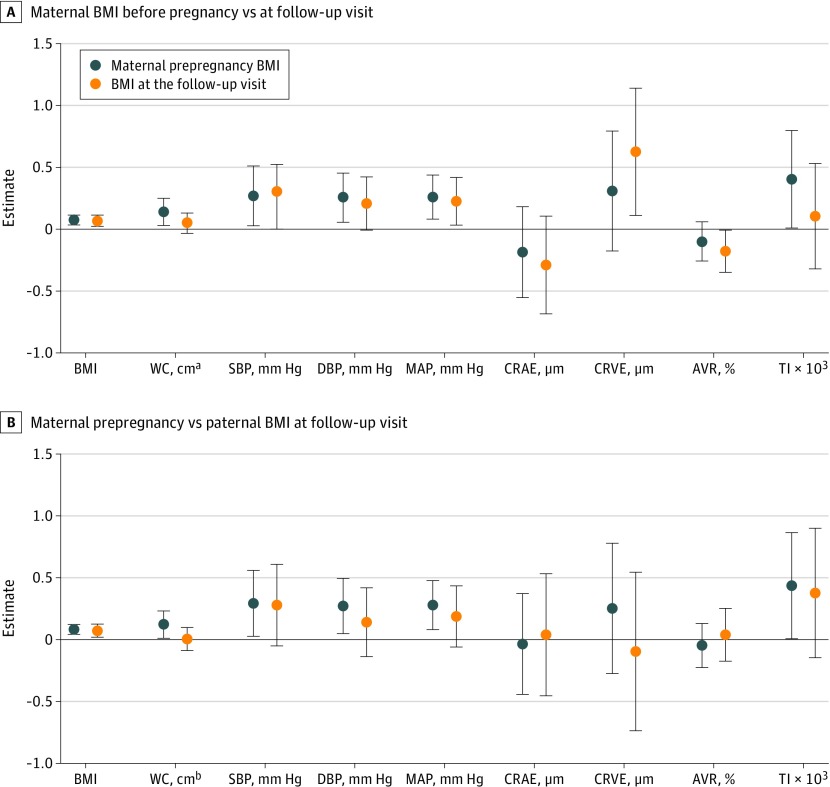
Comparison of Estimates Between Maternal Prepregnancy Body Mass Index (BMI) and Maternal BMI at the Follow-up Visit For 240 Participants and Between Maternal Prepregnancy BMI and Paternal BMI at the Follow-up Visit For 214 Participants Estimates represent the difference (with 95% CIs) in child cardiometabolic outcomes associated with an increase of 1 in maternal prepregnancy BMI (calculated as weight in kilograms divided by height in meters squared; both panels, blue circles) or an increase of 1 in maternal BMI at the follow-up visit (top panel, orange circles) or an increase of 1 in paternal BMI at the follow-up visit (bottom panel, orange circles). Estimates were adjusted for sex, gestational age, parity, newborn race/ethnicity, maternal age, maternal education, maternal smoking, gestational weight gain, date and season of follow-up visit, child’s age, and birth weight. Models for blood pressure and retinal microcirculation parameters were additionally adjusted for children’s BMI, models for retinal parameters were additionally adjusted for MAP, and models for central retinal arteriolar equivalent (CRAE) were additionally adjusted for central retinal venular equivalent (CRVE) and vice versa. Children’s birth weight and BMI were modeled using a natural cubic spline with 3 df. AVR indicates arteriole-to-venule ratio; DBP, diastolic blood pressure; MAP, mean arterial pressure; SBP, systolic blood pressure; TI, tortuosity index; WC, waist circumference. ^a^N = 237. ^b^N = 211.

The assumption of a linear association between maternal prepregnancy BMI and children’s cardiometabolic outcomes was reasonable for children’s BMI, blood pressure parameters, and TI (eFigures 3 and 4 in the Supplement). However, the positive associations with children’s birth weight and WC appeared to flatten out (birth weight) or become negative (WC) for maternal prepregnancy BMI values above the median (23.4).

## Discussion

The key findings of this study are: (1) mothers that were heavier before pregnancy had children with higher blood pressure at age 4 to 6 years and (2) independent from the association with children’s BMI and blood pressure, maternal prepregnancy BMI was positively associated with children’s retinal vessel tortuosity. These associations could not be explained by the positive associations observed between prepregnancy BMI and children’s anthropometry. To our knowledge, our study is the first to assess the association between maternal prepregnancy BMI and their children’s retinal microvascular traits.

Our findings might reflect shared familial-genetic factors or lifestyle habits as well as fetal programming mechanisms. Associations with maternal BMI at the follow-up visit were slightly weaker than those for prepregnancy BMI (except for children’s retinal vessel width parameters) and associations with paternal BMI were not significant (except for children’s BMI), supporting the hypothesis of direct intrauterine mechanisms. A plausible mechanism is that fetal overnutrition affects the development of hypothalamic pathways that regulate energy expenditure and appetite pathways, thereby predisposing the offspring to cardiometabolic disease.^30^

In a recent review on the association between maternal prepregnancy BMI and offspring blood pressure, only 5 of 16 studies on this subject were rated as good-quality research with adequate adjustment for covariates, such as maternal age, maternal smoking, maternal socioeconomic status, offspring’s sex, and offspring’s age.^11^ The authors concluded that the association between maternal prepregnancy BMI and offspring’s blood pressure is likely mediated via the offspring’s anthropometry because after additional adjustment for anthropometric factors, a significant association between maternal prepregnancy BMI and offspring’s blood pressure was only reported in 2 studies.^31,32^ Both studies involved children age 5 or 6 years and the adjustment for children’s birth weight and BMI resulted in a decrease in effect estimates of nearly 50%, largely caused by the correction for children’s BMI. Lawlor and colleagues^31^ found a significant association between maternal prepregnancy BMI and child’s SBP (β = 0.17 and β = 0.09 mm Hg before and after adjustment for children’s anthropometry respectively) and Gademan and colleagues^32^ reported a significant association with children’s DBP (β = 0.11 and β = 0.07 mm Hg before and after adjustment for children’s anthropometry respectively). The estimates in the current study are considerably larger and only decreased slightly (SBP: β = 0.34 and β = 0.27 mm Hg) or even increased (DBP: β = 0.17 and β = 0.25 mm Hg) after adding child’s birth weight and BMI to the model. This is likely because of the lack of association between children’s birth weight and blood pressure in our study and the absence of an association between children’s BMI and blood pressure at BMI values above the median (15.9). For child BMI values below the median, we observed a slightly positive association with child SBP, and, unexpectedly, a negative association with DBP, while many studies indicate a positive association between BMI and blood pressure in children.^33,34,35^ Maternal prepregnancy BMI has also been associated with other cardiometabolic risk factors in the offspring, such as total body and abdominal fat mass measures, insulin levels, glucose levels, homeostatic model assessment of insulin resistance, triglycerides, and high-density lipoprotein cholesterol levels.^7,36,37,38^ However, these associations were also largely explained by current offspring BMI.

A higher maternal prepregnancy BMI was associated with an increased retinal TI in the offspring. Significant associations with child CRVE and AVR were only observed for maternal BMI measured at the follow-up visit (positive and negative association, respectively). Child CRVE was also independently associated with the birth weight and BMI of the child, with significant increases in CRVE at higher birth weights and BMI values. Wider retinal venules in children with a high BMI have also been observed in other studies,^39,40,41,42^ often accompanied with narrower retinal arterioles.^39,40,41^ Obesity in children has also been reported to be associated with a higher venular curvature tortuosity.^43^

Measures of microvascular status have been associated with several cardiovascular risk factors in adults^44,45^ as well as children.^46^ In British elderly individuals, metabolic risk factors were associated with increased tortuosity and width of retinal venules, whereas atherosclerotic risk factors were associated more closely with arteriolar width (negative association).^44^ In a multiethnic population of 10-year-old children in the UK, retinal arteriolar tortuosity was positively associated with levels of triglyceride, total and low-density lipoprotein cholesterol, and SBP and DBP.^46^ Alterations in retinal microvasculature may be causal to the development and/or aggravation of components of the metabolic syndrome through several interrelated mechanisms. Microvascular changes because of hypertension may cause a reduction in insulin sensitivity by reducing the skeletal muscle uptake of glucose, whereas microvascular changes resulting from excessive adiposity may increase peripheral vascular resistance and, consequently, blood pressure.^45^

### Limitations

A limitation of our study is that although mother-newborn pairs from ENVIRONAGE are representative for the general Flemish population,^21^ the sample size with follow-up data are still limited. Nevertheless, the average values for anthropometric, blood pressure, and retinal vessel parameters are similar to those observed in other healthy child populations of the same age.^31,32,33,47^ Our findings do not necessarily reflect causal associations and may be because of unmeasured factors associated with maternal BMI and offspring cardiometabolic outcomes. However, residual confounding is unlikely given the prospective collection of detailed covariate information from early pregnancy onwards and the extensive adjustment for confounders, such as maternal education, maternal smoking, gestational weight gain, and children’s birth weight and BMI. Another limitation is the lack of offspring adiposity indicators other than BMI and WC. Although BMI has been strongly criticized for not distinguishing fat from lean mass, recent studies suggest that BMI is associated with good or even better predictions for later-life cardiometabolic traits and cardiovascular mortality than more expensive fat mass measurements.^48,49^ We also did not have data on other cardiometabolic biomarkers at age 4 to 6 years, such as insulin, glucose, cholesterol, and inflammation levels. However, the availability of retinal microcirculation measurements in young children is unique to this study and enabled us to investigate, to our knowledge for the first time, changes in the microvasculature associated with maternal prepregnancy BMI. Moreover, these retinal images were analyzed by 1 researcher, limiting examiner bias in these analyses. Given that self-reported weight tends to be underreported, another strength of this study is the availability of actual maternal weight measured at the first antenatal visit and maternal and child weight measured at the follow-up visit. However, paternal weight was self-reported, which might have led to an underestimation of associations with paternal BMI.

The public health relevance of our findings is high given that CVD is the leading cause of death worldwide^50^ and a growing body of evidence suggests that adult CVD risk may be set during early life. Childhood BMI and blood pressure are associated with adult cardiovascular health,^51,52,53^ and early-life subclinical microvascular changes may be involved in the future development of CVD. Although the predictive value of early-life retinal imaging for adult cardiovascular health is still unclear, cross-sectional studies suggest that changes in the retinal microvasculature are associated with cardiometabolic risk factors in children,^54^ and adult studies have shown that retinal microcirculation parameters are predictive for cardiovascular disease.^14,15,17,18^

## Conclusions

We found that anthropometric measurements, blood pressure parameters, and retinal vessel tortuosity in children age 4 to 6 years were significantly associated with maternal prepregnancy BMI. Associations with child cardiovascular outcomes were not significant for paternal BMI and were independent of children’s BMI, suggesting that direct intrauterine mechanisms may be involved. Although future studies should examine the causality and underlying mechanisms of the observed associations, our findings add to the evidence that a healthy maternal weight before conception may reduce the CVD risk of the next generation.

## Notes

Supplement. **eTable.** Characteristics of the ENVIRONAGe sample compared to all births in Flanders **eFigure 1.** The shape of the associations between birth weight or child’s BMI and blood pressure parameters **eFigure 2.** The shape of the associations between birth weight or child’s BMI and retinal microcirculation parameters **eFigure 3. **The shape of the associations between maternal pre-pregnancy BMI and child’s birth weight, BMI, waist circumference, and blood pressure parameters **eFigure 4. **The shape of the associations between maternal pre-pregnancy BMI and child’s retinal microcirculation parameters
