# Supplementary material for: Association Between Maternal Prepregnancy Body Mass Index and Anthropometric Parameters, Blood Pressure, and Retinal Microvasculature in Children Age 4 to 6 Years
Source: JAMA Netw Open. 2020 May 12;3(5):e204662. doi: 10.1001/jamanetworkopen.2020.4662 (PMC7218490; doi:10.1001/jamanetworkopen.2020.4662)
Supplement: Supplement. — eTable. Characteristics of the ENVIRONAGe sample compared to all births in Flanders eFigure 1. The shape of the associations between birth weight or child’s BMI and blood pressure parameters eFigure 2. The shape of the associations between birth weight or child’s BMI and retinal microcirculation parameters eFigure 3. The shape of the associations between maternal pre-pregnancy BMI and child’s birth weight, BMI, waist circumference, and blood pressure parameters eFigure 4. The shape of the associations between maternal pre-pregnancy BMI and child’s retinal microcirculation parameters [file jamanetwopen-3-e204662-s001.pdf]

Supplementary Online Content

Cox B, Luyten LJ, Dockx Y, et al. Association between maternal prepregnancy body mass index and anthropometric parameters, blood pressure, and retinal microvasculature in children age 4 to 6 years. *JAMA Netw Open*. 2020;3(5):e204662. 10.1001/jamanetworkopen.2020.4662

eTable. Characteristics of the ENVIRONAGe sample compared to all births in Flanders

eFigure 1. The shape of the associations between birth weight or child's BMI and blood pressure parameters

eFigure 2. The shape of the associations between birth weight or child's BMI and retinal microcirculation parameters

eFigure 3. The shape of the associations between maternal pre-pregnancy BMI and child's birth weight, BMI, waist circumference, and blood pressure parameters

eFigure 4. The shape of the associations between maternal pre-pregnancy BMI and child's retinal microcirculation parameters

This supplementary material has been provided by the authors to give readers additional information about their work.

© 2020 Cox et al. *JAMA Network Open.*

eTable. Characteristics of the ENVIR*ON*AGE sample compared to all births in Flanders.

	**ENVIR*ON*AGE sample (n=240)**	**All deliveries Flanders (n=606877)§**
Mother		
Age, *years*	29·9 (25·0-35·0)	29·5 (23·5-35·8)
Parity		
1	50·8	46·9
2	37·9	34·7
≥3	11·3	18·4
Maternal educational level		
Low	7·1	13·1
Middle	26·7	40·8
High	66·3	46·1
Newborn		
Boys	47·5	51·4
Gestational age, *weeks*	39·2 (37-41)	38·9 (37-41)
Mild preterm (34-36 wks)	4·6	4·8
Moderate preterm (32-33 wks)	0·4	0·6
Extreme preterm (<32 wks)	0·0	0·6
Birth weight, *g*	3445 (2879-4046)	3360 (2740-3965)
European ethnicity	93·8	87·7

Values are percentages or means (10th-90th percentiles).

§2002-2011 (Cox et al., 2013).[1]

© 2020 Cox et al. *JAMA Network Open.*

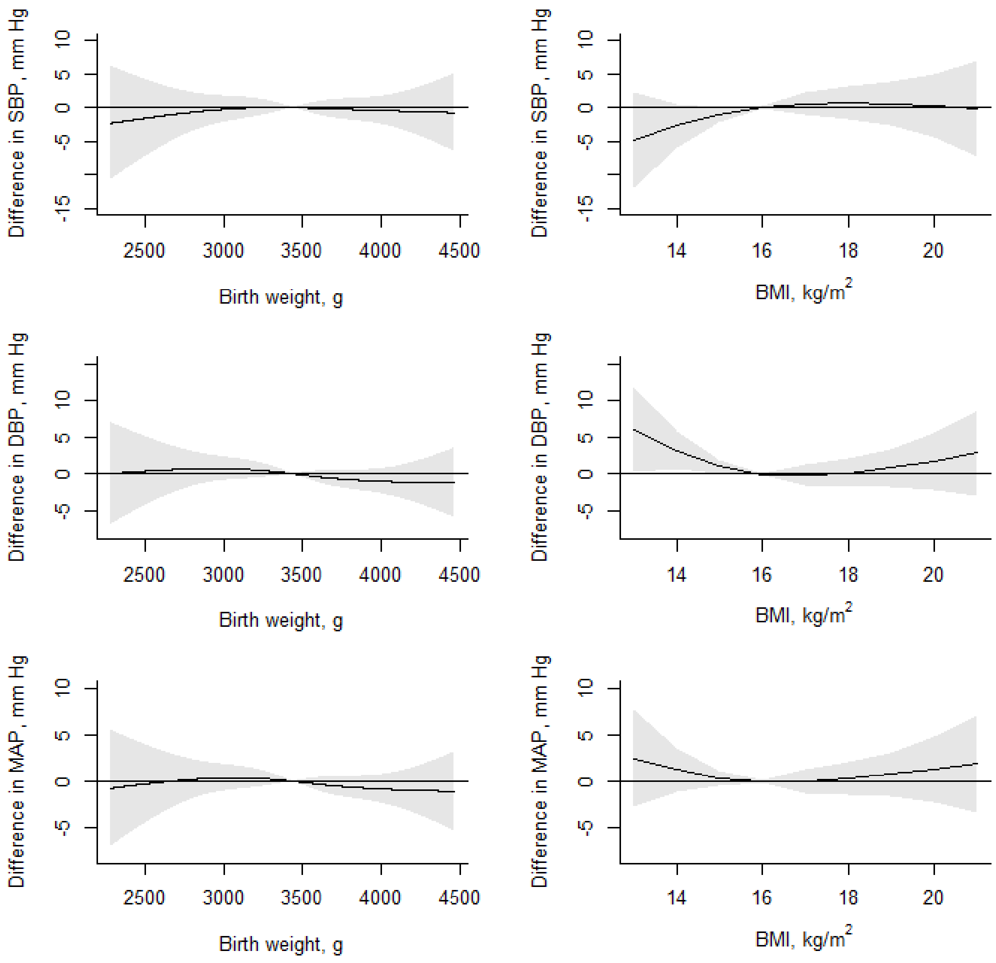

eFigure 1. The shape of the associations between child's birth weight (left) or BMI (right) and blood pressure parameters.

Estimates represent the difference in blood pressure (with 95% CI) relative to the median birth weight (3440 g) or BMI (15·9 kg/m^2) and were adjusted for sex, gestational age, parity, child's ethnicity, maternal age at delivery, maternal education, maternal smoking, maternal pre-pregnancy BMI, gestational weight gain, date and season of follow-up visit, and child's age. Estimates for child's birth weight were adjusted for child's BMI and vice versa. Child's birth weight and BMI were modeled using a natural cubic spline with 3 degrees of freedom. Abbreviations: CI, confidence interval; BMI, body mass index; SBP, systolic blood pressure; DBP, diastolic blood pressure; MAP, mean arterial pressure.

© 2020 Cox et al. *JAMA Network Open.*

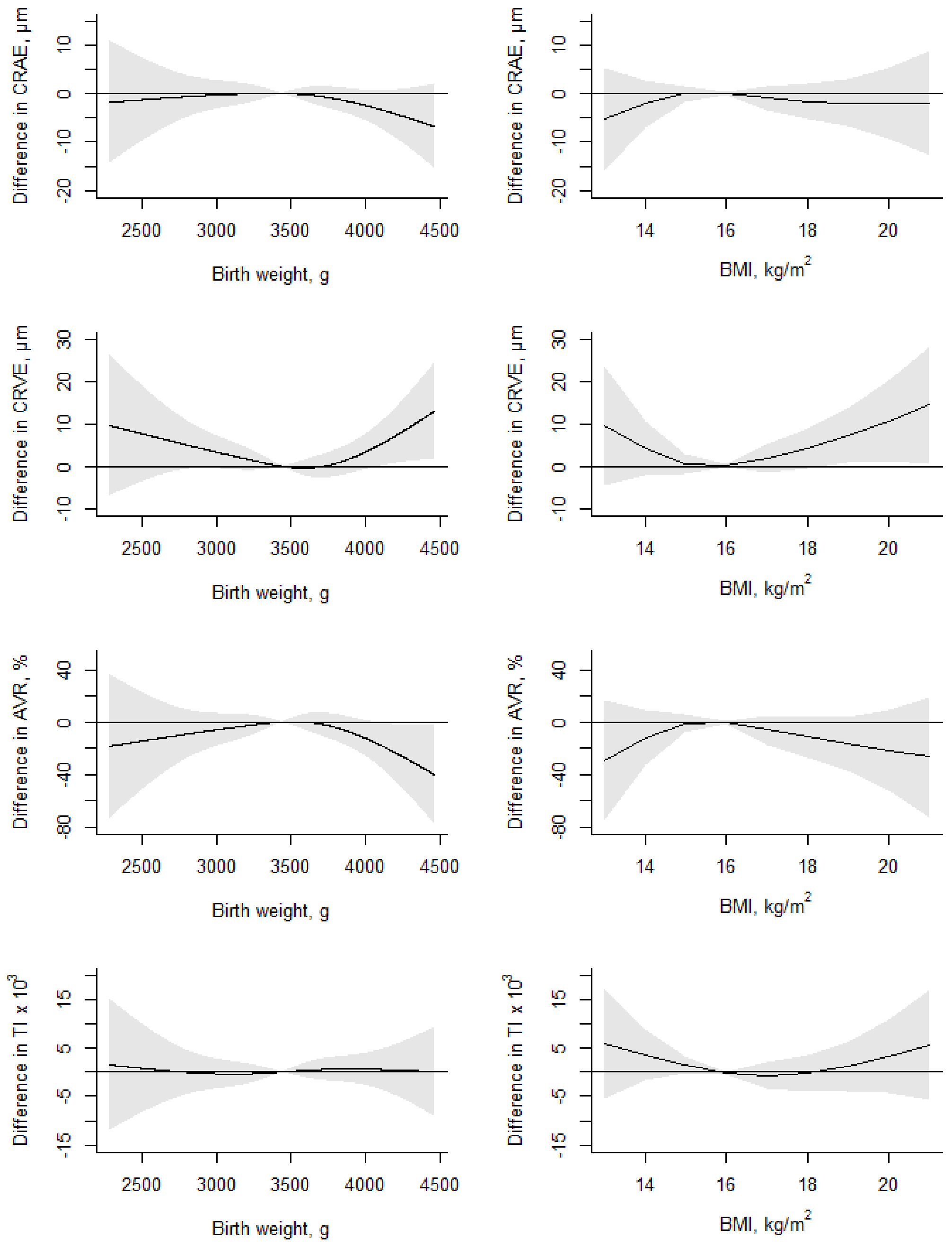

eFigure 2. The shape of the associations between child's birth weight (left) or BMI (right) and retinal microcirculation parameters.

Estimates represent the difference in retinal microcirculation (with 95% CI) relative to the median birth weight (3440 g) or BMI (15·9 kg/m^2) and were adjusted for sex, gestational age, parity, child's ethnicity, maternal age at delivery, maternal education, maternal smoking, maternal pre-pregnancy BMI, gestational weight gain, date and season of follow-up visit, child's age, and MAP. Estimates for child's birth weight were adjusted for child's BMI and vice versa. Estimates for CRAE were additionally adjusted for CRVE and vice versa. Child's birth weight and BMI were modeled using a natural cubic spline with 3 degrees of freedom. Abbreviations: CI, confidence interval; BMI, body mass index; MAP, mean arterial pressure; CRAE, central retinal arteriolar equivalent; CRVE, central retinal venular equivalent; AVR, arteriole-to-venule ratio; TI, tortuosity index.

© 2020 Cox et al. *JAMA Network Open.*

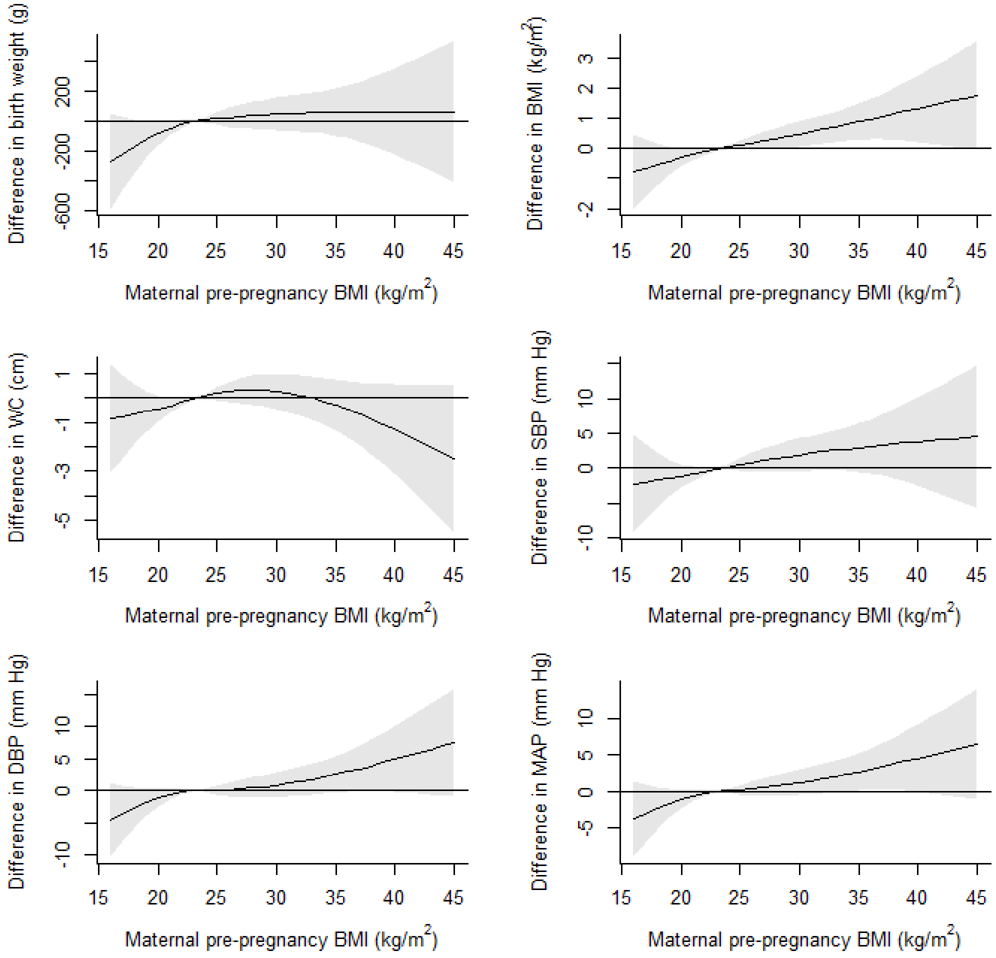

eFigure 3. The shape of the associations between maternal pre-pregnancy BMI and child's birth weight, BMI, waist circumference, and blood pressure parameters.

Estimates represent the difference in outcomes (with 95% CI) relative to the median maternal pre-pregnancy BMI (23·4 kg/m^2) and were adjusted for sex, gestational age, parity, child's ethnicity, maternal age at delivery, maternal education, maternal smoking, gestational weight gain, date and season of follow-up visit, child's age, birth weight (except for the model for birth weight), and BMI (except for the model for birth weight, BMI, and WC). Child's birth weight, BMI, and maternal pre-pregnancy BMI were modeled using a natural cubic spline with 3 degrees of freedom. Abbreviations: CI, confidence interval; BMI, body mass index; WC, waist circumference; SBP, systolic blood pressure; DBP, diastolic blood pressure; MAP, mean arterial pressure.

© 2020 Cox et al. *JAMA Network Open.*

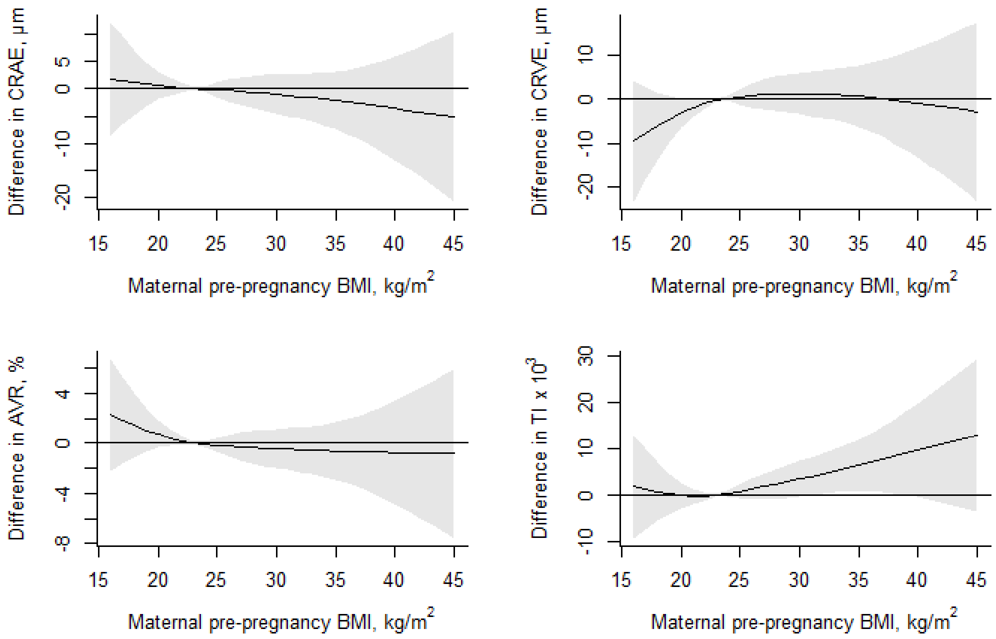

eFigure 4. The shape of the associations between maternal pre-pregnancy BMI and child's retinal microcirculation parameters.

Estimates represent the difference in outcomes (with 95% CI) relative to the median maternal pre-pregnancy BMI (23·4 kg/m^2) and were adjusted for sex, gestational age, parity, child's ethnicity, maternal age at delivery, maternal education, maternal smoking, gestational weight gain, date and season of follow-up visit, child's age, birth weight, BMI, and MAP. Estimates for CRAE were additionally adjusted for CRVE and vice versa. Child's birth weight, BMI, and maternal pre-pregnancy BMI were modeled using a natural cubic spline with 3 degrees of freedom. Abbreviations: CI, confidence interval; BMI, body mass index; MAP, mean arterial pressure; CRAE, central retinal arteriolar equivalent; CRVE, central retinal venular equivalent; AVR, arteriole-to-venule ratio; TI, tortuosity index.

© 2020 Cox et al. *JAMA Network Open.*

References

1. Cox B, Martens E, Nemery B, Vangronsveld J, Nawrot TS. Impact of a stepwise introduction of smoke-free legislation on the rate of preterm births: analysis of routinely collected birth data. *BMJ.* 2013;346:f441.

© 2020 Cox et al. *JAMA Network Open.*
